# A Changing of the Guard: Immune Checkpoint Inhibitors With and Without Chemotherapy as First Line Treatment for Metastatic Non-small Cell Lung Cancer

**DOI:** 10.3389/fonc.2019.00195

**Published:** 2019-03-29

**Authors:** Jose M. Pacheco, D. Ross Camidge, Robert C. Doebele, Erin Schenk

**Affiliations:** Division of Medical Oncology, Department of Internal Medicine, University of Colorado Cancer Center, Aurora, CO, United States

**Keywords:** NSCLC, checkpoint inhibitors, clinical trials, KEYNOTE, CheckMate, IMpower

## Abstract

Inhibitory antibodies targeting programmed death protein 1 (PD-1) and programmed death ligand 1 (PD-L1) have resulted in improved outcomes for many patients with metastatic non-small cell lung cancer in (NSCLC) in the second-line setting due to their ability to lead to prolonged anti-tumor immune responses. Combining these immunotherapies with platinum-based chemotherapy as first-line treatment has resulted in improved response rates and increased survival when compared to platinum-based chemotherapy alone. Certain patient populations may even benefit from immune checkpoint inhibitors as monotherapy in the first-line setting. The PD-1 inhibitor pembrolizumab is approved as monotherapy or in combination with platinum + pemetrexed for most newly diagnosed patients with metastatic NSCLC, excluding those with a targetable oncogene such as ALK and EGFR. The PD-L1 inhibitor atezolizumab is also approved in combination with bevacizumab + carboplatin + paclitaxel for the same population, with some parts of the world also approving this regimen for patients with ALK rearrangements or EGFR activating mutations. However, there are many other chemo-immunotherapy regimens that have been evaluated as initial treatment in metastatic NSCLC. Additionally, combinations of PD-1 axis inhibitors with cytotoxic T lymphocyte antigen-4 inhibitors have been examined, although none are yet approved. Here we review the clinical data in support of the current first-line approaches across histologies and biomarker subtypes, as well as highlight future research directions revealed by the current data.

## Introduction

Since approval of nivolumab as second line therapy for metastatic squamous non-small cell lung cancer (NSCLC) in 2015, first line treatment options for NSCLC have rapidly evolved to include checkpoint inhibitors (1). Under normal conditions, the immune checkpoints programmed death protein 1 (PD-1), programmed cell death ligand 1 (PD-L1), and cytotoxic T lymphocyte antigen-4 (CTLA-4) are best understood as controls for activated T cells that limit their subsequent detection and responsiveness to antigen (2). Checkpoint inhibitors that block PD-1 (nivolumab and pembrolizumab) or PD-L1 (atezolizumab, durvalumab, and avelumab) prevent T cell downregulation initiated by PD-1 binding PD-L1 expressed on tumor cells and immune cells (3). Ipilimumab and tremelimumab prevent the interaction of CTLA-4 on T cells with CD80 or CD86 on antigen presenting cells, allowing CD28, the co-receptor necessary for effective T cell stimulation, to bind (4). Currently, a PD-1 axis inhibitor is recommended as first line therapy alone or in combination with chemotherapy for most patients with metastatic NSCLC, excluding those with a targetable oncogene such as *ALK* and *EGFR* (1). Here we review the clinical data in support of the current approaches across histologies and biomarker subtypes, as well as highlight the future research directions revealed by the current data.

## Methods

Trials were identified by searching PubMed without date limits, clinicaltrials.gov and abstracts/presentations from major medical society meetings since 2015 (American Association for Cancer Research annual meeting, American Society of Clinical Oncology annual meeting, European Society of Medical Oncology annual meeting and the International Association on Study of Lung Cancer World Conference on Lung Cancer). Trials not reporting data specific to NSCLC were excluded.

The following search terms were used: KEYNOTE-024, KEYNOTE-042, CheckMate 026, IMpower132, IMpower130, IMpower131, KEYNOTE-407, osimertinib + durvalumab, gefinitib + durvalumab, atezolizumab plus erlotinib, pembrolizumab + gefitinib, pembrolizumab and *EGFR*, pembrolizumab + erlotinib, nivolumab + crizotinib, alectinib + atezolizumab, KEYNOTE-021, duration PD-1 lung cancer, *EGFR* pembrolizumab, *EGFR* nivolumab, *EGFR* atezolizumab, *EGFR* durvalumab, *EGFR* avelumab, *ALK* pembrolizumab, *ALK* nivolumab, *ALK* atezolizumab, *ALK* durvalumab, and *ALK* avelumab. This article conforms to the Committee on Publication Ethics (COPE) and the International Committee on Medical Journal Editors (ICMJE) recommendations on ethics.

### First Line Immune Checkpoint Inhibitors Without Chemotherapy

#### Pembrolizumab

Pembrolizumab has been compared to platinum-based doublets as first line systemic therapy in two randomized phase III trials in patients independent of histology and negative for *EGFR* activating mutations or *ALK* rearrangements. KEYNOTE-024 enrolled patients with PD-L1 ≥50% and KEYNOTE-042 enrolled patients with PD-L1 ≥1% (5–8).

KEYNOTE-024 led to pembrolizumab becoming an integral part of first line treatment for the approximately 30% of patients with NSCLC who express PD-L1 ≥50% on tumor cells and lacking *EGFR* activating mutations or *ALK* rearrangements (5). This trial demonstrated improvements in objective response rate (ORR), progression free survival (PFS), and overall survival (OS) with pembrolizumab when compared to chemotherapy (Table 1). Notably, improved OS with pembrolizumab monotherapy was observed despite a 62.3% crossover rate at time of progression on chemotherapy to pembrolizumab. Grade ≥3 treatment related adverse events (TRAEs) were less with pembrolizumab at 31.2% vs. 53.3% with chemotherapy (5–7). This trial resulted in the United States Food and Drug Administration (FDA) approval of pembrolizumab as a first line monotherapy option for patients with any histology NSCLC and PD-L1 ≥ 50% (1, 9).

**Table 1:** Phase 3 trials of checkpoint inhibitors alone or in combination for first line treatment of metastatic NSCLC.

**Trial**	**Histology**	PD-L1 staining^*^	**Therapy**	**ORR (95% CI)**	**Median PFS (95% CI)**	**Median OS (95% CI)**
KEYNOTE-024 (5, 7)	Squamous and non-squamous	≥50%	Pembrolizumab (*n* = 154)	45.5% (37.4–53.7)	10.3 mo (6.7-NR)	30 mo (18.3-NR)
			Platinum Doublet (*n* = 151)	29.8% (22.6–37.8)	6.0 mo (4.2–6.2)	14.2 mo (9.8–19.0)
KEYNOTE-042 (8)	Squamous and non-squamous	≥50%	Pembrolizumab (*n* = 299)	39.5%^a^	7.1 mo (5.9–9.0)	20 mo (15.4–24.9)
			Platinum doublet (*n* = 300)	32.0%^a^	6.4 mo (6.1–6.9)	12.2 mo (10.4–14.2)
		1–49%	Pembrolizumab (*n* = 338)	16.6% (12.8–21.0)	N/A	13.4 (10.7–18.2)
			Platinum Doublet (*n* = 337)	21.7% (17.4–26.4)	N/A	12.1 (11.0–14.0)
CheckMate-026 (10)	Squamous and non-squamous	≥50%	Nivolumab (*n* = 88)	34% (24.0–45.0)	5.4^a^	15.9^a^
			Platinum doublet (*n* = 126)	39% (30.0–48.0)	5.8^a^	13.9^a^
		≥5%	Nivolumab (*n* = 211)	26% (20.0–33.0)	4.2 mo (3.0–5.6)	14.4 mo (11.7–17.4)
			Platinum doublet (*n* = 212)	33% (27.0–40.0)	5.9 mo (5.4–6.9)	13.2 mo (10.7–17.4)
CheckMate-227 (11)	Squamous and non-squamous	Any	Nivolumab + Ipilimumab (*n* = 583)	N/A	4.9 mo (4.1–5.6)	N/A
			Platinum doublet (*n* = 583)	N/A	5.5 mo (4.6–5.6)	N/A
MYSTIC (12)	Squamous and non-squamous	≥50%	Durvalumab (*n* = 118)	N/A	N/A	18.3 mo (13.6–22.8)
			Durvalumab + Tremelimumab (*n* = 108)	N/A	N/A	15.2 mo (8.0–26.5)
			Platinum doublet (*n* = 107)	N/A	N/A	12.7 mo (10.3–15.1)
		≥25%	Durvalumab (*n* = 163)	35.6%^a^	4.7 mo (3.1–6.3)	16.3 mo (12.2–20.8)
			Durvalumab + Tremelimumab (*n* = 163)	34.4%^a^	3.9 mo (2.8–5.0)	11.9 mo (9.0–17.7)
			Platinum doublet (*n* = 162)	37.7%^a^	5.4 mo (4.6–5.8)	12.9 mo (10.5–15.0)

* *PD-L1 staining on tumor cells was defined by the 22C3 assay for pembrolizumab, the Dako 28-8 assay for nivolumab and the SP263 assay for durvalumab. Platinum includes either carboplatin or cisplatin*.

a *Confidence interval not available*.

*PD-L1, programmed death ligand 1; ORR, objective response rate; PFS, progression free survival; OS, overall survival; CI, confidence interval; mo, months; NR, not reached; NE, not evaluable; N/A, not available*.

Similarly, KEYNOTE-042 demonstrated improved OS with pembrolizumab, HR 0.81 (95%CI, 0.71–0.93) and *p* = 0.0018 (Table 1) (8). However, these OS results need to be interpreted with caution as nearly half of patients had PD-L1 ≥50%. For patients with PD-L1 of 1–49% OS was not improved with pembrolizumab monotherapy vs. chemotherapy, HR 0.92 (95%CI, 0.77–1.11). In agreement with KEYNOTE-024, patients with PD-L1 ≥50% experienced an improved OS with pembrolizumab. In patients with PD-L1 ≥1%, the ORR and PFS were similar between the two arms. As reported in KEYNOTE-024, pembrolizumab was better tolerated than chemotherapy (8). Pembrolizumab monotherapy is not recommended by the National Comprehensive Cancer Network (NCCN) guidelines or FDA approved for first line treatment in patients with PD-L1 <50% (1, 9).

#### Nivolumab

Nivolumab monotherapy does not have a clear role in first line therapy despite its success in the second line setting for immunotherapy-naïve patients. CheckMate-026 was a randomized phase III trial comparing nivolumab to platinum-based doublets as initial systemic therapy in patients with PD-L1 ≥5% (10). Enrolled patients had squamous or non-squamous NSCLC without activating *EGFR* mutations or *ALK* rearrangements. In contrast to the trials with pembrolizumab monotherapy, nivolumab did not demonstrate improvement in any major trial endpoint compared to chemotherapy even when enriching for PD-L1 expression at a level potentially comparable to that in KEYNOTE-024 (Table 1). In patients with PD-L1 ≥5%, the PFS HR was 1.15 (95%CI, 0.91–1.45) and OS HR was 1.02 (95%CI, 0.80–1.30), and similarly, patients with PD-L1 ≥50% experienced no improvement with nivolumab monotherapy by PFS or OS (1.07 (95%CI, 0.77–1.49) and 0.90 (95%CI, 0.63–1.29), respectively) (10). Based on the absence of improved outcomes compared to chemotherapy, nivolumab as monotherapy is neither FDA approved nor listed in the NCCN guidelines for first line treatment of metastatic NSCLC (1, 9).

#### Nivolumab + Ipilimumab

Dual checkpoint blockade with nivolumab + ipilimumab was initially tested as first line therapy in CheckMate-012, a single arm phase I trial for patients with any histology NSCLC and any degree PD-L1 staining (13). Nivolumab 3 mg/kg every 2 weeks and ipilimumab 1 mg/kg every 6 weeks resulted in an encouraging ORR and 2-year OS, especially in patients with PD-L1 ≥50% (13, 14). Based on these results, a randomized phase III study (CheckMate-227) compared nivolumab + ipilimumab to platinum-based chemotherapy in both PD-L1 positive and PD-L1 negative patients without activating *EGFR* mutations or *ALK* rearrangements. In the overall trial population of CheckMate227, nivolumab + ipilimumab modestly improved 1 year PFS compared to platinum-based doublets, HR 0.83 (95%CI, 0.72–0.96) but did not improve median PFS or OS (Table 1). The incidence of grade 3–4 TRAEs were similar with nivolumab + ipilimumab at 31.2% when compared to platinum-based doublets at 36.1%. Nivolumab + ipilimumab is not in the NCCN guidelines or FDA approved for use in NSCLC (1, 9).

#### Durvalumab + Tremelimumab

The MYSTIC trial evaluated first line durvalumab ± tremelimumab compared to platinum-based doublets (12). Patients of any histology NSCLC with any PD-L1 expression who lacked *EGFR* activating mutations or *ALK* rearrangements were enrolled. None of the prespecified primary endpoints for patients with PD-L1 expression ≥25% were met (Table 1). In those patients, durvalumab and tremelimumab did not improve PFS or OS compared to chemotherapy, HR 1.05 (95%CI, 0.72–1.53) and HR 0.85 (95%CI, 0.61–1.17), respectively. Similarly, durvalumab monotherapy did not improve OS compared to chemotherapy in patients with PD-L1 ≥ 25% [HR 0.76 (95%CI, 0.56–1.02)] (12). Even in exploratory analysis, patients with PD-L1 ≥50% experienced no significant improvements in OS with either immunotherapy regimen (Table 1). No new safety signals emerged in the immunotherapy arms and TRAEs were less compared to platinum-based doublets (12). This combination is not part of NCCN guidelines or FDA approved for patients with NSCLC (1, 9).

#### Avelumab

A phase Ib single arm study evaluated avelumab in patients regardless of PD-L1 staining levels who lacked *EGFR* activating mutations or *ALK* rearrangements (15). The ORR was 18.7% and the median PFS was 2.71 months (95% CI, 1.56–4.18). A phase III trial is forthcoming randomizing patients of any histology NSCLC to avelumab alone or histology directed platinum doublets (NCT02576574).

### First Line Immune Checkpoint Inhibition With Chemotherapy

#### Non-squamous Histology

##### Pembrolizumab + platinum (carboplatin or cisplatin) + pemetrexed

Due to low or absent PD-L1 tumor expression, the majority of patients with metastatic non-squamous NSCLC are not eligible for pembrolizumab monotherapy. Patients with PD-L1 < 50% and those with PD-L1 ≥50% were enrolled in KEYNOTE-189, which compared the combination of pembrolizumab + platinum + pemetrexed to platinum + pemetrexed. Patients with activating *EGFR* mutations or *ALK* rearrangements were excluded. Platinum and pemetrexed were administered together for 4 cycles, followed by maintenance pemetrexed. Pembrolizumab was given every 3 weeks for up to 35 cycles (16, 17).

All efficacy endpoints were improved with the addition of pembrolizumab to chemotherapy across PD-L1 subgroups: PD-L1 negative, PD-L1 1–49% and PD-L1 ≥50%, except for PFS in PD-L1 negative patients (Table 2) (16, 17). For the entire cohort, the ORR was 47.6% with pembrolizumab + chemotherapy and 18.9% with chemotherapy (HR not provided, *p* < 0.001). Triplet therapy improved PFS and OS vs. chemotherapy, (HR 0.52, *p* < 0.001 and HR 0.49, *p* < 0.01, respectively). Notably, for the PD-L1 ≥50% subgroup, the benefits of pembrolizumab + chemotherapy were more pronounced. The ORR was 61.4% with pembrolizumab + chemotherapy (*n* = 132) vs. 22.9% (*n* = 70) with chemotherapy (*p* < 0.0001). The PFS and OS were also prolonged with pembrolizumab + chemotherapy (HR 0.36 (95% CI 0.25–0.52) and HR 0.42 (95% CI 0.24–0.68), respectively) (Table 2) (16, 17). Pembrolizumab plus platinum and pemetrexed for first line management of non-squamous NSCLC is listed in the NCCN guidelines and FDA approved regardless of the PD-L1 staining level (1, 9).

**Table 2:** Phase 3 trials of checkpoint inhibitors plus chemotherapy for first line treatment of metastatic non-squamous NSCLC.

**Trial**	PD-L1 staining^*^	**Therapy**	**ORR (95% CI)**	**Median PFS (95% CI)**	**Median OS (95% CI)**	**1 year OS (95% CI)**
KEYNOTE-189 (16, 17)	TC ≥ 50%	Pembrolizumab + Platinum + Pemetrexed (*n* = 132)	61.4% (52.5–69.7)	9.4 mo (9.0–13.8)	NR	73%^a^
		Platinum + Pemetrexed (*n* = 70)	22.9% (13.7–34.4)	4.7 mo (3.1–6.0)	10.0 mo (7.5-NE)	48.1%^a^
	Any	Pembrolizumab + Platinum + Pemetrexed (*n* = 410)	47.6% (42.6–52.5)	8.8 mo (7.6–9.2)	NR	69.2% (64.1–73.8)
		Platinum + Pemetrexed (*n* = 206)	18.9% (13.8–25.0)	4.9 mo (4.7–5.5)	11.3 mo (8.7–15.1)	49.4% (42.1–56.2)
IMpower150 (18–20)	TC ≥ 50% or IC ≥ 10%^b^	Atezolizumab + Bevacizumab + Carboplatin + Paclitaxel (*n* = 71)	69%^a^	12.6 mo (10.9–23.4)	25.2^a^	N/A
		Bevacizumab + Carboplatin + Paclitaxel (*n* = 64)	49%^a^	6.8 mo (5.6–8.4)	15.0^a^	N/A
	Any	Atezolizumab + Bevacizumab + Carboplatin + Paclitaxel (*n* = 356)	63.5% (58.2–68.5)	8.3 mo (7.7–9.8)	19.2 mo (18.0–23.8)	67.3% (62.4–72.2)
		Bevacizumab + Carboplatin + Paclitaxel (*n* = 336)	48% (42.5–53.6)	6.8 mo (6.0–7.1)	14.7 mo (13.3–16.9)	60.6% (55.3–65.9)
IMpower130 (21)	TC ≥ 50% or IC ≥ 10%^b^	Atezolizumab + Carboplatin + Nab-paclitaxel (*n* = 88)	N/A	6.4 mo (5.49–9.76)	17.3 mo (14.78-NR)	N/A
		Carboplatin + Nab-paclitaxel (*n* = 42)	N/A	4.6 mo (3.22–7)	16.0 mo (10.94-NR)	N/A
	Any	Atezolizumab + Carboplatin + Nab-paclitaxel (*n* = 451)	49.2%^a^	7.0 mo (6.2–7.3)	18.6 mo (16–21.2)	63.1%^a^
		Carboplatin + Nab-paclitaxel (*n* = 228)	31.9%^a^	5.5 mo (4.4–5.9)	13.9 mo (12.0–18.7)	55.5%^a^
IMpower132 (22)	TC ≥ 50% or IC ≥ 10%^b^	Atezolizumab + Platinum + Pemetrexed (*n* = 25)	72%^a^	10.8 mo^a^	N/A	N/A
		Platinum + Pemetrexed (*n* = 20)	55%^a^	6.5 mo^a^	N/A	N/A
	Any	Atezolizumab + Platinum + Pemetrexed (*n* = 292)	47%^a^	7.6 mo (6.6–8.5)	18.1 mo (13.0-NE)	N/A
		Platinum + Pemetrexed (*n* = 286)	32%^a^	5.2 mo (4.3–5.6)	13.6 mo (11.4–15.5)	N/A

* *PD-L1 staining on tumor cells was defined by the 22C3 assay for pembrolizumab and the Dako 28-8 assay for nivolumab. With atezolizumab PD-L1 staining on tumor cells or immune cells was done using the SP142 assay. Platinum includes either carboplatin or cisplatin*.

a *Confidence interval not available*.

b *For the IMpower studies patients with PD-L1 ≥ 50% on tumor cells or PD-L1 ≥ 10% immune cells are grouped together as PD-L1 high staining*.

*PD-L1, programmed death ligand 1; ORR, objective response rate; PFS, progression free survival; OS, overall survival; CI, confidence interval; mo, months; NR, not reached; NE, not evaluable; TC, tumor cells; IC, immune cells; N/A, not available*.

The addition of pembrolizumab in this trial resulted in a minimal increase in the overall adverse event rate when compared to chemotherapy (grade ≥3 in 67.2% vs. 65.8%) and this did not appear to differ significantly by the type of platinum used. As expected, the immune mediated adverse event rate was higher with the addition of pembrolizumab (all grades 22.7% vs. 11.9%, grade ≥3 in 8.9% vs. 4.5%). With regards to any etiology adverse event, diarrhea, and rash were significantly more common with the addition of pembrolizumab (diarrhea: all grades 30.9% vs. 21.3% and grade ≥3 in 5.2% vs. 3.0%; rash: all grades 20.2% vs. 11.4%, grade ≥3 in 1.7% vs. 1.5%). Additionally, incidence of neutropenic fever was greater with pembrolizumab; however, overall incidence of this was low (16, 17).

##### Atezolizumab + bevacizumab + carboplatin + paclitaxel

Atezolizumab is the only other checkpoint inhibitor with a first line approval in metastatic NSCLC based on IMpower150 which compared atezolizumab + bevacizumab + carboplatin + paclitaxel (ABCP) to bevacizumab + carboplatin + paclitaxel (BCP) in patients with any level PD-L1. Chemotherapy + bevacizumab was administered for 4–6 cycles. Bevacizumab ± atezolizumab was administered every 3 weeks until disease progression or death (18).

In contrast to most other studies, patients with activating *EGFR* mutations or *ALK* rearrangements were allowed to enroll if they had progressed on or were unable to tolerate at least one tyrosine kinase inhibitor (TKI) but excluded from the primary end point assessment. All efficacy endpoints were improved with ABCP vs. BCP (Table 2) (18–20). Across all PD-L1 subgroups, ABCP significantly improved PFS compared to BCP. As noted in other immunotherapy trials, patients with tumor PD-L1 expression ≥50% or, unique to atezolizumab PD-L1 assessment, immune cell PD-L1 expression ≥10%, had a greater magnitude of benefit with the addition of atezolizumab (Table 2) (18–20). ABCP is listed in the NCCN guidelines for first line therapy in patients with advanced non-squamous NSCLC, as well as for patients with EGFR activating mutations or ALK rearrangements who have progressed on at least one prior TKI. While FDA approved for front line therapy in patients with advanced non-squamous NSCLC, the FDA approval does not include patients with *EGFR* activating mutations or *ALK* rearrangements (1, 9).

The addition of atezolizumab in this trial resulted in no difference in the incidence of any grade TRAEs, but an increase in grade 3–4 TRAEs was seen (55.7% vs. 45.7%). Immune mediated adverse events occurred with a greater frequency with the addition of atezolizumab. Similar to what was seen in KEYNOTE-189, the addition of atezolizumab lead to a higher incidence of rash and febrile neutropenia (rash: grade 1–2 in 12.0% vs. 5.1% and grade 3–4 in 1.3% vs. 0%; febrile neutropenia: grade 1–2 in 0.5% vs. 0%, grade 3–4 in 8.4% vs. 5.8%, and grade 5 in 0.8% vs. 0%) (18–20).

##### Atezolizumab + carboplatin + nab-paclitaxel

IMpower130, a key corollary to IMpower150, compared atezolizumab + carboplatin + nab-paclitaxel to carboplatin + nab-paclitaxel in patients regardless of PD-L1 staining, including patients with activating *EGFR* mutations or *ALK* rearrangements after 1st line TKI. Carboplatin + nab-paclitaxel was administered for 4–6 cycles. Patients receiving chemotherapy alone were treated with either placebo or pemetrexed every 3 weeks. Atezolizumab was administered every 3 weeks until disease progression or death (21).

As in IMpower150, enrolled *EGFR*, or *ALK*+ patients were not included in the primary analysis. Adding atezolizumab to chemotherapy improved PFS and OS in the entire trial population (Table 2) (21). Subgroup analyses based on PD-L1 levels observed a PFS improvement regardless of PD-L1 expression, but none experienced a significant OS benefit with the addition of atezolizumab. Grade ≥3 TRAEs were 74.9% with atezolizumab + chemotherapy vs. 60.7% with chemotherapy (21). This regimen is neither FDA approved nor in the NCCN guidelines for non-squamous NSCLC (1, 9).

##### Atezolizumab + platinum (carboplatin or cisplatin) + pemetrexed

IMpower132, an ongoing phase III trial, is testing the chemotherapy backbone from KEYNOTE-189 with atezolizumab in patients with any level PD-L1 excluding those with activating *EGFR* mutations or *ALK* rearrangements. Platinum + pemetrexed was given for 4–6 cycles, followed by pemetrexed maintenance. Atezolizumab was administered every 3 weeks until disease progression or death (22).

Similar to KEYNOTE-189, the ORR and PFS were improved when the checkpoint inhibitor atezolizumab was added to chemotherapy (Table 2). However, at the interim analysis, the OS for the entire cohort was not prolonged with the addition of atezolizumab to chemotherapy, HR 0.81 (95%CI, 0.64–1.03) (22). In early subgroup analysis, patients with tumor PD-L1 ≥50% or immune cell PD-L1 ≥10% appear to have a greater magnitude of benefit with the addition of atezolizumab (Table 2) (22). This regimen is neither FDA approved nor in the NCCN guidelines for non-squamous NSCLC (1, 9).

#### Squamous Histology

##### Pembrolizumab + carboplatin + taxane (nab-paclitaxel or paclitaxel)

Extrapolating from the success of adding pembrolizumab to frontline chemotherapy in non-squamous histology, KEYNOTE-407 compared carboplatin + a taxane with (*n* = 278) or without pembrolizumab (*n* = 281) in patients with squamous histology and any level PD-L1. Carboplatin + taxane was administered for 4 cycles. Pembrolizumab was given every 3 weeks for up to 35 cycles (23, 24).

All efficacy endpoints were improved with the addition of pembrolizumab to chemotherapy across PD-L1 subgroups: PD-L1 negative, PD-L1 1–49%, and PD-L1 ≥50%, except for OS in patients with PD-L1 ≥50% (Table 3). While there was not a statistically significant increase in OS for patients with PD-L1≥50% administered pembrolizumab + chemotherapy (HR 0.64, 95%CI 0.37–1.10), this could reflect the relatively short follow-up and may become statistically significant in the future (23, 24). This combination is in the NCCN guidelines and FDA approved for first line management of squamous histology NSCLC regardless of PD-L1 staining level (1, 9).

**Table 3:** Phase 3 trials of checkpoint inhibitors plus chemotherapy for first line treatment of metastatic squamous NSCLC.

**Trial**	PD-L1 staining^*^	**Therapy**	**ORR (95% CI)**	**Median PFS (95% CI)**	**Median OS (95% CI)**	**1 year OS (95% CI)**
KEYNOTE-407 (23, 24)	TC ≥ 50%	Pembrolizumab + Carboplatin + Paclitaxel or Nab-Paclitaxel (*n* = 103)	60.3% (48.1–71.5)	8.0 mo (6.1–10.3)	NR (11.3-NE)	63.4%^a^
		Carboplatin + Paclitaxel or Nab-Paclitaxel (*n* = 104)	32.9% (22.3–44.9)	4.2 mo (2.8–4.6)	NR (7.4-NE)	51.0%^a^
	Any	Pembrolizumab + Carboplatin + Paclitaxel or Nab-Paclitaxel (*n* = 278)	57.9% (51.9–63.8)	6.4 mo (6.2–8.3)	15.9 mo (13.2-NR)	65.2%^a^
		Carboplatin + Paclitaxel or Nab-Paclitaxel (*n* = 281)	38.4% (32.7–44.4)	4.8 mo (4.3–5.7)	11.3 mo (9.5–14.8)	48.3%^a^
IMpower131 (25)	TC ≥ 50% or IC ≥ 10%^b^	Atezolizumab + Carboplatin + Nab-paclitaxel (*n* = 53)	60%^a^	10.1 mo^a^	23.6 mo^a^	N/A
		Carboplatin + Nab-paclitaxel (*n* = 48)	33%^a^	5.5 mo^a^	14.1mo^a^	N/A
	Any	Atezolizumab + Carboplatin + Nab-paclitaxel (*n* = 343)	49%^a^	6.3 mo (5.7–7.1)	14.0 mo (12.0–17.0)	N/A
		Carboplatin + Nab-paclitaxel (*n* = 340)	41%^a^	5.6 mo (5.5–5.7)	13.9 mo (12.3–16.4)	N/A

* *PD-L1 staining on tumor cells was defined by the 22C3 assay for pembrolizumab. With atezolizumab PD-L1 staining on tumor cells or immune cells was done using the SP142 assay. Platinum includes either carboplatin or cisplatin*.

a *Confidence interval not available*.

b *For the IMpower131 study patients with PD-L1 ≥50% on tumor cells or PD-L1 ≥10% immune cells are grouped together as PD-L1 high*.

*PD-L1, programmed death ligand 1; ORR, objective response rate; PFS, progression free survival; OS, overall survival; CI, confidence interval; mo, months; NR, not reached; NE, not evaluable; TC, tumor cells; IC, immune cells; N/A, not available*.

The addition of pembrolizumab led to no significant increase in the overall adverse event rate, grade ≥3 in 69.8% vs. 68.2%. Immune mediated adverse events and infusion reactions were more common in patients receiving pembrolizumab (all grades 28.8% vs. 8.6%, grade ≥3 in 10.3% vs. 3.2%). Alopecia (all grades 46.0% vs. 36.4%) and puritis were higher with the addition of pembrolizumab; however, there was no significant increase in the incidence of diarrhea, rash or febrile neutropenia (23, 24).

##### Atezolizumab + carboplatin + nab-paclitaxel

IMpower131, an ongoing phase III trial, is comparing carboplatin + nab-paclitaxel with (*n* = 343) or without atezolizumab (*n* = 340) regardless of PD-L1 status. Chemotherapy was administered for 4 or 6 cycles. Atezolizumab was given every 3 weeks until disease progression or death (25).

At the prespecified interim analysis, the addition of atezolizumab to chemotherapy improved ORR. While atezolizumab + chemotherapy prolonged PFS, it did not improve OS (HR 0.96, 95%CI 0.78–1.18) (Table 3) (26). As reported in the other IMpower trials, patients with high PD-L1 (tumor cells ≥50% or immune cells ≥10%) experienced a greater magnitude of benefit with addition of atezolizumab to chemotherapy (Table 3). Grade ≥3 TRAEs were 69% with atezolizumab + chemotherapy vs. 58% with chemotherapy (25). This regimen is neither FDA approved nor in the NCCN guidelines for squamous histology NSCLC (1, 9).

### Immune Checkpoint Inhibitors for Oncogene Addicted NSCLC

#### First Line Checkpoint Inhibitor Monotherapy

As checkpoint inhibitors emerged as a viable therapeutic option for NSCLC and other malignancies, multiple approaches attempted to incorporate checkpoint inhibitors alone or in combination for the management of NSCLC with an oncogenic driver. Ideally, the non-overlapping mechanisms of action of checkpoint inhibitors and TKIs would result in a deeper or longer duration of response. Early enthusiasm was high as it was common for lung cancers with activating *EGFR* mutations or *ALK* rearrangement to also express PD-L1 and preclinical work demonstrated signaling through *EGFR* or *ALK* upregulated PD-L1 expression (27–29). However, results from clinical trials in this patient population have been disappointing.

In treatment naïve NSCLC patients with *EGFR* mutations, a phase II trial of single agent pembrolizumab as first line therapy was closed early due to futility (30). One of 11 patients experienced a partial response, but subsequent tissue analysis revealed non-mutated *EGFR* in the responder. For the patients with *EGFR* mutations, 7 of 10 experienced stable disease as their best response with a median PFS of 6.6 months. Notably, tumor PD-L1 expression was ≥50% in 70% of the patients with documented *EGFR* mutations, suggesting the PD-L1 biomarker is not predictive of benefit with pembrolizumab in patients with *EGFR* activating mutations. Of the seven patients who transitioned to TKI therapy post pembrolizumab, one experienced grade 3 transaminitis resulting in treatment discontinuation and another patient developed grade 5 pneumonitis (30). These concerning safety signals, including after the immunotherapy was completed, and lack of improved efficacy are also emerging from early trials of TKIs in combination with checkpoint inhibitors.

#### First Line Checkpoint Inhibitors Plus TKI

In patients with *EGFR* activating mutations without prior TKI exposure, several trials have reported early safety and efficacy data on *EGFR* directed TKIs in combination with checkpoint inhibitors. A multi-arm phase Ib trial suspended the cohort combining osimertinib and durvalumab due to 7 of the 11 treated patients developing interstitial lung disease (ILD), with three patients experiencing a grade ≥3 ILD (31). Confirmed responses were observed in 70% (95%CI, 35–93) of the patients on combined therapy, a rate similar to first line osimertinib alone (32). In a phase Ib trial, erlotinib combined with atezolizumab demonstrated an ORR of 75% (95%CI, 51–91) and there was a suggestion of a potential PFS benefit with median PFS 15 months (95% CI, 8-not evaluable) (33). However, there were signs of increased toxicity with the erlotinib + atezolizumab combination, with grade 3–4 TRAEs of 46% compared to treatment emergent adverse event rates of 17–45% with erlotinib monotherapy (33–35). Gefitinib combined with durvalumab, generated no new safety signals compared to the TKI alone (36–38). As noted with the osimertinib data, the ORR for gefitinib plus durvalumab or erlotinib plus atezolizumab were similar to rates reported for TKI monotherapy (33–38). Pembrolizumab plus erlotinib or gefitinib resulted in discordant toxicities in a phase I/II trial (39). While the erlotinib combination was similar to erlotinib alone, gefitinib + pembrolizumab resulted in grade 3/4 hepatic toxicity in 5 of the 7 treated patients. Even though it was tolerated, pembrolizumab + erlotinib resulted in a 41.7% ORR, significantly lower than expected with erlotinib monotherapy. However, the PFS appeared to be improved with the combination (39).

For patients with metastatic *ALK*+ NSCLC, a similar theme of toxicity without clear improvement in efficacy has been observed. Cohort E of CheckMate370 evaluated crizotinib with nivolumab in treatment naïve patients and was stopped early after 5 of the first 13 patients developed grade ≥3 hepatic toxicity, 2 of these 5 patients died (40). Efficacy appeared reduced with the combination as the ORR was only 38% compared to the expected ORR of ~65% reported with crizotinib monotherapy in other trials (41). Hepatic and pancreatic toxicities lead to dose reductions of ceritinib in an ongoing phase I trial when combining the TKI with nivolumab (42). Alectinib plus atezolizumab in a phase Ib trial reported a grade ≥3 TRAE rate of 52.4%, a rate higher than expected for alectinib alone (43, 44). The addition of a checkpoint inhibitor to ceritinib or alectinib resulted in an ORR comparable to each TKI as first line monotherapy (44, 45).

Early trials reporting on a combination of TKI and checkpoint inhibitor suggest response rates equivalent to TKI alone and high potential for increased toxicity with these combinations in *EGFR* or *ALK*+ patients. Notably, retrospective data in patients with BRAF mutations suggest a benefit from checkpoint inhibitors in subsequent lines of therapy but no prospective data is available (46). As mature data becomes available for the ongoing trials, measures such as duration of response and time to next therapy will be critical. Whether the increased risk of these combinations is balanced by an efficacy benefit will help determine the direction of future trials, but due to the prolonged benefit seen with TKIs alone the data will likely be uninterpretable in the absence of randomization.

#### Checkpoint Inhibitors Plus Chemotherapy Post TKI Progression

Data has emerged on the combination of chemotherapy plus atezolizumab for advanced NSCLC patients with *EGFR* activating mutations or *ALK* rearrangements previously treated with at least one approved TKI. IMpower150 compared bevacizumab + carboplatin + paclitaxel with or without atezolizumab and enrolled a small subset of *EGFR* or *ALK*+ patients (*n* = 108) (19, 20). *EGFR* or *ALK*+ patients experienced a median PFS of 9.7 months with ABCP compared to 6.1 months with BCP, HR 0.59 (95%CI, 0.37–0.94). OS, while not statistically significant, appears to be trending toward an improvement with ACBP vs. BCP (not reached vs. 17.5 months, HR 0.54 (95%CI 0.29–1.03). Subgroup analyses suggested the benefit in the *EGFR*/*ALK*+ patients may be predominately driven by patients with *EGFR* exon 19 deletions and L858R point mutations in exon 21 (*n* = 59). In contrast to IMpower150, survival for the smaller *EGFR*/*ALK*+ subgroup in IMpower130 was not improved with atezolizumab + carboplatin + paclitaxel (*n* = 32) when compared to carboplatin + paclitaxel (*n* = 12), PFS HR 0.85 (95%CI, 0.36–1.54), and OS HR 0.98 (95%CI, 0.41–2.31) (21). Whether the benefit seen in IMpower150 is due to the addition of bevacizumab to chemotherapy plus atezolizumab or driven by patients with specific molecular alterations or whether some other imbalances existed between the arms to create a spurious result in this small subgroup within IMpower150 will need to be addressed in future trials.

### Selection of First Line Immunotherapy Options by Tumor Mutational Burden

An emerging biomarker of interest for patients with metastatic NSCLC is tumor mutational burden (TMB). TMB is a measure of potential neoantigens that may be recognized by tumor-reactive T cells and it is independent of PD-L1 staining. An initial signal that TMB could predict responsiveness to immune checkpoint inhibitors was seen with nivolumab in CheckMate 026 (10). In a *post-hoc* analysis, patients with a high TMB by whole exome sequencing had a trend toward a better PFS with nivolumab compared to platinum-based chemotherapy, HR 0.62 (95%CI, 0.38–1.00) (4).

A subsequent phase III trial, CheckMate 227, reported on 299 systemic therapy naïve patients with a TMB ≥10 mutations/megabase by the Foundation Medicine targeted sequencing assay (11). Dual immune checkpoint blockade for patients with high TMB resulted in a median PFS of 7.2 vs. 5.5 months for platinum doublet chemotherapy, and at 1 year 43% of patients treated with nivolumab plus ipilimumab had not progressed vs. 13% of patients who received chemotherapy, PFS HR 0.58 (95% CI, 0.41–0.81). Notably, PFS benefit was observed even in patients with high TMB and a PD-L1 level of < 1%, HR 0.48 (95% CI, 0.27–0.85). The PFS improvement did not translate into an OS benefit in the high TMB cohort as the recently updated data for nivolumab plus ipilimumab reported a median OS of 23.03 vs. 16.72 months for chemotherapy alone HR 0.77 (95% CI 0.56–1.06) (47).

Similarly, subgroup analysis suggested high TMB may predict for improved outcomes with durvalumab + tremelimumab when compared to platinum-based doublets (12). The median OS was 16.5 vs. 10.5 months, 2-year OS 39% vs. 18%, HR 0.62 (95% CI, 0.45–0.86). In both trials, patients stopped dual checkpoint blockade due to adverse events at a frequency similar to the rate reported for the discontinuation of pembrolizumab plus a platinum doublet (17, 24). Neither combination of PD-1/PD-L1 axis blockade plus anti-CTLA-4 therapy are in the NCCN guidelines for NSCLC nor FDA approved but may find a place in therapy with mature trial data (1, 9).

## Discussion

Within the past few years, immunotherapy with checkpoint inhibitors has transformed first line therapy for patients with metastatic NSCLC. Prior to this, the most recent update to systemic therapy for newly diagnosed metastatic NSCLC without a driver mutation was the addition of pemetrexed in 2009 (1, 9).

Disrupting the binding of PD-1 to PD-L1 by blocking either partner may be considered equivalent when promoting a T cell response (48). However, the predominance of a PD-1 inhibitor in first line therapy for metastatic NSCLC suggests additional biologic determinants could also dictate a patient's response. Blocking PD-1 prevents inhibition through both PD-L1 and PD-L2, while blocking PD-L1 does not prevent PD-L2 mediated inhibition of T-cell function. Additionally, expression of PD-1, and PD-L1 varies across immune and non-immune cell types and targeting one or the other may result in different subsets of cells responding to the tumor (49). The responding subsets of cells may better synergize with one class of chemotherapy than another based on the specific immune effects of each chemotherapy. Chemotherapy efficacy, while primarily driven by an apoptotic response to damage recognition at the level of a cell's DNA or the cell replication machinery, is also dependent on the immune system (50). Platinum agents, the backbone of chemotherapy for metastatic NSCLC, can increase antigen presentation by cancer cells, promote T cell trafficking into the tumor microenvironment, and decrease local immunosuppressive cells (51, 52). These significant alterations within the tumor microenvironment by platinum agents may not synergize as well with PD-L1 blockade because of the rapidly shifting tumor landscape. In contrast, PD-1 blockade targets a relatively static cell population in comparison, the T cell repertoire. Together these data suggest that better understanding the systemic immune impact of checkpoint inhibitors and chemotherapy are key to developing rational immunotherapy combinations for future trials.

**Figure 1:**
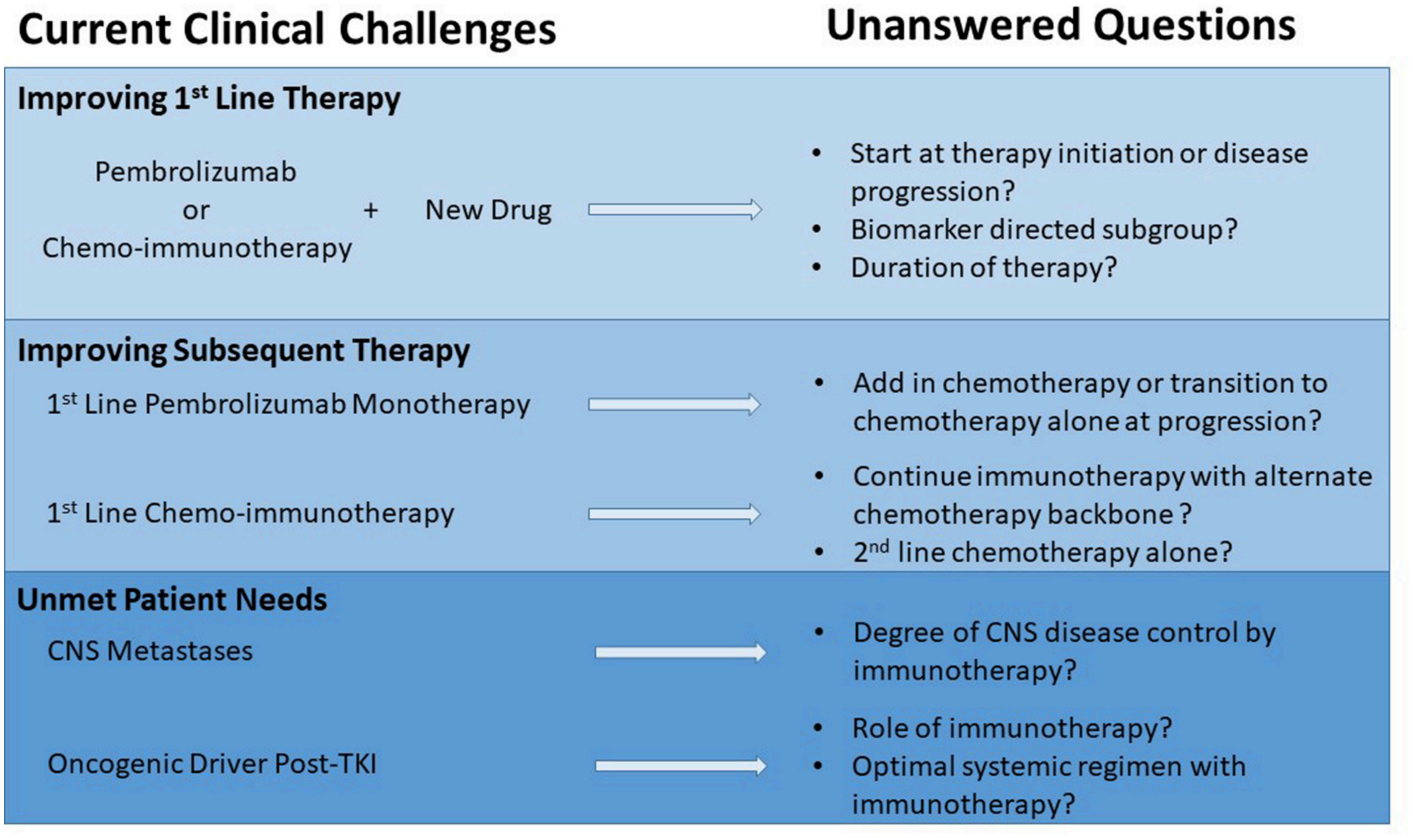
Opportunities in metastatic NSCLC to maximize impact of checkpoint inhibitors.

While still in the formative years of incorporating checkpoint inhibitors into first line therapy for patients with metastatic NSCLC, most of the major trials use chemotherapy as a comparator. Therefore, it is not possible to say with good scientific rigor that a specific chemo-immunotherapy combination is more effective as none of these checkpoint inhibitors alone or in combination have been compared head to head. Several key questions remain about the clinical application of the different regimens and how to build upon the early successes (Figure 1).

Currently available clinical trial data does not clearly establish whether immunotherapy alone or immunotherapy and chemotherapy is the optimal management strategy for patients with PD-L1 ≥50% and no *EGFR* activating mutations or *ALK* rearrangements. For such patients, comparing across studies, the chemo-immunotherapy combinations have a greater ORR compared to pembrolizumab monotherapy (Tables 1, 2). However, cross-trial comparisons may be complicated by the lack of uniform populations being explored, even when grouped by similar minimal levels of PD-L1 expression (7, 8). To date, PFS and OS with chemo-immunotherapy combinations do not yet appear different when compared to pembrolizumab monotherapy (Tables 1, 2). The absence of survival difference may be due to a shorter follow up in the chemo-immunotherapy trials or because of shorter duration of response with chemo-immunotherapy combinations when compared to responses to pembrolizumab alone. Continued translational research is necessary to define patient subsets and biomarkers that may better inform treatment choices for those patients with PD-L1 ≥50%.

Of the biomarkers currently in clinical use, a retrospective single arm study of patients with PD-L1 ≥50% treated with pembrolizumab monotherapy suggested raising the PD-L1 cut-point to ≥90% on tumor cells may enrich for patients who benefit from pembrolizumab monotherapy (53). Patients with ≥90% PD-L1 experienced an ORR of 55% (33 of 60) vs. 25.2% (22 of 87) in patients with 50–89% staining. This cutoff also resulted in improved PFS and OS with a median OS of 33.6 months (95%CI, not reached-not reached) for patients with PD-L1 ≥90% compared to 15.2 months (95%CI, 11.6–25.6) in patients with PD-L1 of 50–89% (53). The power of multiple immune biomarkers was illustrated in a retrospective study across multiple tumor types from 4 KEYNOTE trials (54). While these patients were treated with pembrolizumab in subsequent lines of therapy, a combination of TMB and T cell gene expression profile in the tumor microenvironment enriched for those who responded to immunotherapy and, of equal importance, robustly identified those who did not respond. While encouraging and hypothesis generating, the clear limitation of these retrospective studies is the lack of a comparator reflective of clinical options. Even more challenging, robust predictors for patients who benefit from chemo-immunotherapy have not been identified. While PD-L1 staining appears to have a modest association with outcome, in most studies patients at all PD-L1 levels, including PD-L1 negative patients, appear to benefit. Clearly, continued collection, and study of biospecimens from all patients starting immunotherapy alone or in combination is a priority to improve patient selection for therapies and inform trials testing drug combinations with immunotherapy.

In the absence of clear data, patient performance status and clinical scenario often drive the selection of pembrolizumab monotherapy or combined chemo-immunotherapy for those with PD-L1 ≥50%. While pembrolizumab monotherapy is much better tolerated compared to available chemo-immunotherapy combinations, it is worth noting that historically 1/3 to 1/2 of patients do not receive second line therapy (55). For the first time in the immune checkpoint inhibitor era, available initial therapy options and subsequent therapy sequencing will be tested in a randomized phase III trial, the INSIGNA study. This trial is planned to enroll 800 patients with any histology metastatic NSCLC with PD-L1 ≥1% (stratified by whether levels are ≥50% or less) and will be randomized to one of three arms. One arm will treat patients with pembrolizumab plus histology directed chemotherapy (56). The two other arms will start patients on pembrolizumab monotherapy but at time of progression one will transition to chemotherapy alone and the other will transition to chemotherapy plus continued pembrolizumab. This trial will be the first head to head comparison between pembrolizumab monotherapy and chemo-immunotherapy and may provide clearer guidance on how to manage patients after progression on pembrolizumab monotherapy, an urgent clinical need. What is not readily addressed by the INSIGNA trial is how to manage patients post-progression on chemo-immunotherapy. Standard of care may include docetaxel ± ramucirumab or other chemotherapies (e.g., gemcitabine). However, these standard treatments generally have short survival benefit. An important future question is whether the checkpoint inhibitor should be continued in this setting but with an alternative chemotherapy backbone.

Future approaches to improve responses and outcomes for patients who start immunotherapy with or without chemotherapy are pembrolizumab combinations with a novel drug as initial treatment and several trials in this paradigm are ongoing in patients with PD-L1 ≥50% (Table 4). Most of these trials are in phase I or II of testing and not all are randomized or use pembrolizumab alone as a comparator arm so definitive answers are likely years away. Similar trials are underway for pembrolizumab + chemotherapy (Table 4).

**Table 4:** Select ongoing first line trials for patients with metastatic NSCLC and PD-L1 ≥50% on tumor cells.

**Regimen**	**Study design**	**NCT number**	**Primary outcome**	**Estimated completion date**
Pembrolizumab + Decitabine + Tetrahydrouridine	Single arm phase I/II	NCT03233724	MTD and ORR	December 31, 2020
Pembrolizumab + Itacitinib	Single arm phase II	NCT03425006	ORR at 12 weeks and toxicity	June 2021
Pembrolizumab + AGEN1884	Single arm phase II	NCT03411473	DLT incidence	May 2021
Pembrolizumab + GRN1201/sargramostim	Single arm phase II	NCT03417882	ORR	March 2021
Pembrolizumab + AM0010 vs. Pembrolizumab (Cypress 1)	Randomized phase II	NCT03382899	ORR	December 2021
Pembrolizumab + Ipilimumab vs. Pembrolizumab + Placebo (KEYNOTE-598)	Randomized phase III	NCT03302234	PFS and OS	February 22, 2024
Pembrolizumab + IO102 vs. Pembrolizumab and Pembrolizumab + Carboplatin + Pemetrexed + IO102 vs. Pembrolizumab + Carboplatin + Pemetrexed	Randomized phase I/II	NCT03562871	DLT incidence, ORR	February 2022
Pembrolizumab + Carboplatin + Pemetrexed + NEO-PV-01/Adjuvant	Phase I	NCT03380871	DLT incidence	February 2021
Pembrolizumab + Platinum + Pemetrexed + Canakinumab vs. Pembrolizumab + Platinum + Pemetrexed (CANOPY-1)	Phase III	NCT03631199	DLT incidence, PFS, and OS	October 21, 2022

*Decitabine is a histone deacetylase inhibitor. Tetrahydrouridine is a cytidine deaminase inhibitor. Itacitinib is a JAK1 inhibitor. AGEN1884 is a CTLA4 inhibitor. GRN1201/sargramostim is a cancer peptide vaccine. AM0010 is a recombinant interleukin 10. Ipilimumab is a CTLA4 inhibitor. IO102 is an IDO inhibitor. NEO-PV-01/Adjuvant is a personalized cancer vaccine and poly-ICLC, a dsRNA. Canakinumab is a monoclonal antibody that neutralizes IL-1β. Platinum includes either carboplatin or cisplatin. PD-L1, programmed death ligand 1; NCT, national clinical trials; ORR, objective response rate; PFS, progression free survival; OS, overall survival; DLT, dose limiting toxicity; MTD, maximum tolerated dose*.

For patients with prolonged PFS on first line pembrolizumab or chemo-immunotherapy, duration of treatment has been dictated by the development of toxicity or disease progression. While early data in patients with metastatic melanoma suggest highly selected patients can stop checkpoint inhibition and maintain response, less is known about patients with metastatic NSCLC. In a small randomized study, patients with NSCLC on second line nivolumab for at least 1 year were randomized to continue nivolumab (*n* = 76) or stop therapy (*n* = 87) in CheckMate-153. PFS favored continuous therapy (HR 0.42, 95% CI 0.25–0.71), but OS was not significantly different (57). While outcome data favored continuous therapy, over half of patients on observation alone continued to experience a response of stable disease or better with a median follow up of 14.9 months. Conclusions are difficult to draw and apply to first line therapy, but it highlights the recurring theme that a cohort of patients experience prolonged, durable benefit from checkpoint inhibitors and efficacy is not completely dependent on scheduled dosing. While a challenging prospect based on the range of patient response and need for continued therapy, future trials addressing maintenance dosing after first line immunotherapy ± chemotherapy are important for patient quality of life and reducing long-term toxicities.

Several groups of patients with metastatic NSCLC have not yet benefitted from immunotherapy either due to poor responses or lack of inclusion in trials. Early data in never smoking patients have suggested immunotherapy alone does not significantly improve PFS or OS over chemotherapy (5–7, 10). Thus, regardless of PD-L1 staining levels, caution may need to be utilized in this patient population before administering immune checkpoint inhibition without chemotherapy as first line treatment. In these patients, proper molecular testing should be done to exclude targetable oncogenic drivers, for which approved TKIs would be the preferred initial therapy.

The role of chemo-immunotherapy combinations after progression on TKIs is not clear since the data from both IMpower130 and IMpower150 was based on small subset analyses in patients with *EGFR* activating mutations/*ALK* rearrangements. This lack of clarity is reflected in the differing approvals by governing bodies, with the FDA not approving ABCP for patients with EGFR activating mutations/ALK rearrangements, and other areas of the world (e.g., the European Medicines Agency) approving ABCP for patients with these genetic subsets of NSCLC. IMpower130, with a subgroup of 44 patients, suggests chemotherapy plus immunotherapy does not benefit *EGFR*/*ALK*+ patients which is in line with nearly all prospective and retrospective studies trying to find a role for monotherapy with immune checkpoint inhibitors in this patient cohort (21). IMpower150 included 108 patients with *EGFR*/*ALK*+ disease and reported a PFS improvement with the addition of bevacizumab to the IMpower130 regimen, especially in the *EGFR*+ patients with an exon 19 deletion or L858R point mutation (18, 19). Bevacizumab has been shown to reduce Treg accumulation in tumors and attenuate Treg expansion in the peripheral blood (58). One interpretation is that multiple alterations of local and systemic immunity are necessary for checkpoint inhibition to benefit patients with a targetable oncogenic driver. The discrepancies between the IMpower studies may be clarified with a phase II trial which will randomize never smokers or those with an *EGFR, ALK*, or *ROS1* driver after at least 1 TKI to carboplatin + pemetrexed + bevacizumab with or without atezolizumab (Table 5). In part, the potential for specific molecular alterations being more likely to respond to immune checkpoint inhibition plus chemotherapy will be addressed in KEYNOTE-789 (Table 5). This phase III trial will randomize patients with an *EGFR* exon 19 deletion or *EGFR* L858R point mutation in exon 21 to pemetrexed and a platinum ± pembrolizumab after progression on an *EGFR* directed TKI. CheckMate722 will test nivolumab + a platinum doublet chemotherapy and nivolumab +ipilimumab against standard chemotherapy for patients with *EGFR* activating mutations who have progressed after at least one TKI. While data for KEYNOTE-789, CheckMate722, and other planned trials will likely inform clinical practice, for now it is unclear where chemotherapy plus immunotherapy fits, if at all, into the therapy schema of patients with targetable oncogenic drivers (Table 5).

**Table 5:** Select chemo-immunotherapy trials for chemotherapy Naïve NSCLC patients with *EGFR* activating mutations or *ALK* rearrangements.

**Regimen**	**Patient population**	**Study design**	**NCT number**	**Primary outcome**	**Estimated completion date**
Atezolizumab + Carboplatin + Pemetrexed + Bevacizumab vs. Carboplatin + Pemetrexed + Bevacizumab	Non-smokers (<100 cigarettes in a life time), *EGFR* activating mutation, or *ALK* or *ROS1* rearrangement positive	Randomized phase II	NCT03786692	PFS	January 2024
Nivolumab + Carboplatin + Pemetrexed vs. Nivolumab + Ipilimumab	*EGFR* activating mutation or *ALK* rearrangement positive	Randomized phase II	NCT03256136	ORR	October 31, 2024
Pembrolizumab + Platinum + Pemetrexed vs. Platinum + Pemetrexed (KEYNOTE-789)	*EGFR* activating mutation positive	Randomized phase III	NCT03515837	PFS and OS	June 26, 2023
Nivolumab + Platinum + Pemetrexed vs. Nivolumab + Ipilimumab vs. Platinum + Pemetrexed (CheckMate722)	*EGFR* activating mutation positive	Randomized phase III	NCT02864251	PFS	December 31, 2023

*Platinum includes either carboplatin or cisplatin. PD-L1, programmed death ligand 1; NCT, national clinical trials; ORR, objective response rate; PFS, progression free survival; OS, overall survival*.

Finally, an estimated 26% of newly diagnosed stage IV NSCLC patients present with brain metastases and very few patients with untreated CNS disease were enrolled on the trials that have redefined first line therapy for metastatic NSCLC (59). In patients with metastatic NSCLC receiving pembrolizumab as a subsequent line of therapy, a small phase II study enrolled patients with limited, asymptomatic brain metastases who either were untreated or progressed after local therapy (60). One third of patients with PD-L1 positive tumors and CNS disease experienced a response while no responses were observed in patients with PD-L1 negative or unevaluable PD-L1 status. Additionally, early trial data from metastatic melanoma has suggested that CNS responses can be achieved with immune checkpoint inhibitors alone in first line therapy (60, 61). With these initial safety and efficacy signals, future first line immunotherapy trials should include this cohort of lung cancer patients to better understand the scope of CNS disease control by PD-1 vs. PD-L1 checkpoint inhibitors given the potential different cell distribution of the respective drug targets and their location relative to the blood brain barrier. Additionally, the role of PD-L1 expression in predicting response to these drugs in untreated CNS disease needs to be better evaluated.

## Conclusion

In the span of only a few years, immunotherapy has transformed the treatment of patients with metastatic NSCLC. In cohorts with high PD-L1 and no targetable oncogene, survival is approaching years rather than months with the previous chemotherapy standard of care. Remarkably, the pairing of chemotherapy and checkpoint inhibition is benefitting patients without high PD-L1 to a similar degree. While the wait for success in the field of cancer immunotherapy and for patients with metastatic NSCLC has been long, these accomplishments should be a building block for developing future therapies and clinical trials. Multiple clinical challenges remain to maximize patient benefit from currently available therapy and continued translational research is necessary to develop rational frameworks in which to test the next generation of immunotherapy.

## Author Contributions

JP and ES identified relevant clinical trials and wrote the initial draft of the manuscript. JP, ES, DC and RD contributed to manuscript revision, read, and approved the submitted version.

### Conflict of Interest Statement

The authors declare that the research was conducted in the absence of any commercial or financial relationships that could be construed as a potential conflict of interest.
